# Single‐ or two‐visits endodontic treatment: Impact of irrigants on surface roughness, microhardness, topography, and fracture resistance of root dentin

**DOI:** 10.1111/eos.70103

**Published:** 2026-05-06

**Authors:** Yasmin Padoin, Sidnei Flores de Pellegrin, Duvan Cala Castillo, William Lemos Bevilaqua, Gabriel Kalil Rocha Pereira, Guilherme Pauletto

**Affiliations:** ^1^ Graduate Program in Dentistry, Faculty of Dentistry Federal University of Santa Maria (UFSM) Santa Maria Rio Grande do Sul Brazil; ^2^ Post‑Graduate Program in Oral Science, Faculty of Dentistry Federal University of Santa Maria (UFSM) Santa Maria Rio Grande do Sul Brazil; ^3^ Industrial Technical College of Santa Maria (CTISM) Federal University of Santa Maria (UFSM) Santa Maria Rio Grande do Sul Brazil

**Keywords:** chlorhexidine, properties of dentin, root canal therapy, sodium hypochlorite

## Abstract

This study evaluated the effect of the number of simulated endodontic treatment visits and the type of irrigant on the surface roughness, microhardness, topography, and fracture resistance of root dentin. Bovine roots were distributed into six groups (*n* = 14) according to the irrigant and number of treatment visits: 0.9% saline solution (single‐ or two‐visits); 2.5% sodium hypochlorite (NaOCl) (single‐ or two‐visits); 2% chlorhexidine (CHX) (single‐ or two‐visits). Next, the roots were embedded in cylindrical acrylic molds for fracture resistance testing and fracture pattern analysis. In addition, dentin blocks were subjected to the same experimental protocols (*n* = 7) and analyzed for surface roughness, topography, and microhardness. Two‐way anova, Tukey's post hoc and Fisher's exact tests were performed. There was no difference between the groups for fracture resistance or failure pattern. For surface roughness, highest values were observed when 2.5% NaOCl was used in combination with two simulated visits, findings that corroborate the dentin erosion observed in the microscopic analysis. For dentin microhardness, 2% CHX resulted in a reduction in this property, whereas the number of visits did not affect the outcome. In conclusion, different irrigants caused microstructural changes in dentin under simulated single‐ and two‐visits treatments, but fracture resistance remained unaffected.

## INTRODUCTION

The anatomical complexity of the root canal system and the microbial load associated with endodontic infections demand careful selection of both the mechanical preparation technique and the chemical agents employed [1, 2]. Even with adequate mechanical instrumentation, a considerable portion of the canal walls may remain uninstrumented, potentially compromising disinfection and treatment success [3, 4]. To overcome this limitation, the use of irrigating solutions is essential as a complementary procedure, given the complex anatomy of the root canal system—marked by the presence of branches, isthmuses, accessory canals, and dentinal tubules [5, 6].

An irrigating solution for use as the main irrigant during endodontic treatment must present as its main attributes a potent antimicrobial action and tissue dissolution capacity [6]. Sodium hypochlorite (NaOCl) remains the most widely used irrigant in endodontics. This chlorine‐based oxidizing agent has a high pH and promotes protein degradation and acid neutralization, exerting antimicrobial action through irreversible oxidation of microbial cellular components [7, 8, 9]. However, its main drawbacks include chemical instability and high cytotoxicity [10, 11]. Alternatively, chlorhexidine (CHX) is a chemically stable biguanide solution with lower cytotoxicity, satisfactory antimicrobial efficacy, and an important advantage—its high substantivity, which allows prolonged antimicrobial activity through interaction with bacterial cell membranes, increasing membrane permeability and leading to cell lysis [7, 12, 13]. These characteristics support the use of CHX as an alternative primary irrigant to NaOCl in endodontic treatment. However, it is generally considered a secondary option because it lacks tissue‐dissolving properties, which limits its clinical applicability [14].

Besides the choice of the main irrigant, another important factor in achieving adequate disinfection of the root canal system is the irrigation time and volume, which are inherently associated with whether endodontic treatment is performed in a single‐ or two‐visits approach [15, 16]. Increasing the number of treatment visits generally increases both the duration and volume of irrigant contact with the dentin structure [17]. From this perspective, there is a lack of studies simultaneously assessing the influence of the type of irrigant and the number of visits on dentin microstructure, as well as the impact of these factors on the fracture resistance of endodontically treated teeth. Investigating these variables is crucial to understanding the longevity of treated teeth and developing strategies to minimize potential adverse effects [18]. The relevance of this analysis lies in the fact that root dentin is a key structural component for tooth survival, enabling the dissipation of stresses generated during mastication and other functional activities, thus preventing the formation of microcracks and fractures [19, 20].

Given the above, the objective of this study was to evaluate the effect of the number of simulated endodontic treatment visits (single‐visit vs. two‐visits) and the type of irrigant (saline solution vs. NaOCl vs. CHX) on the surface roughness, microhardness, topography, and fracture resistance of root dentin. The null hypotheses were that (I) the type of irrigant and (II) the number of simulated endodontic treatment visits would not influence any of the evaluated outcomes.

## MATERIAL AND METHODS

### Sample selection

This study did not require approval from a research ethics committee, as it was an in vitro experiment using bovine teeth that would be discarded. The sample size was calculated using the OpenEpi tool (http://www.openepi.com/SampleSize/SSMean.htm), based on the main outcome of this study (fracture resistance) and data from a similar study [21]. The calculation considered a compressive strength of 105.88 MPa (±28.13) for dentin irrigated with an inert solution and 79.85 MPa (±18.34) for dentin irrigated with NaOCl, with a statistical power of 80% and a significance level of 5%. Based on these parameters, a minimum of 14 samples per group was required. Therefore, for the six experimental groups, a total of 84 teeth were needed. Eighty‐four bovine incisors with complete root development and a single‐root canal were selected. Teeth presenting an apical foramen larger than a #60 file, visible cracks or fractures, or any structural anomalies were excluded. All specimens were examined under a stereomicroscope (Discovery V20; Carl‐Zeiss) at 10× magnification to support the selection process.

### Preoperative evaluation of roots

**FIGURE 1:**
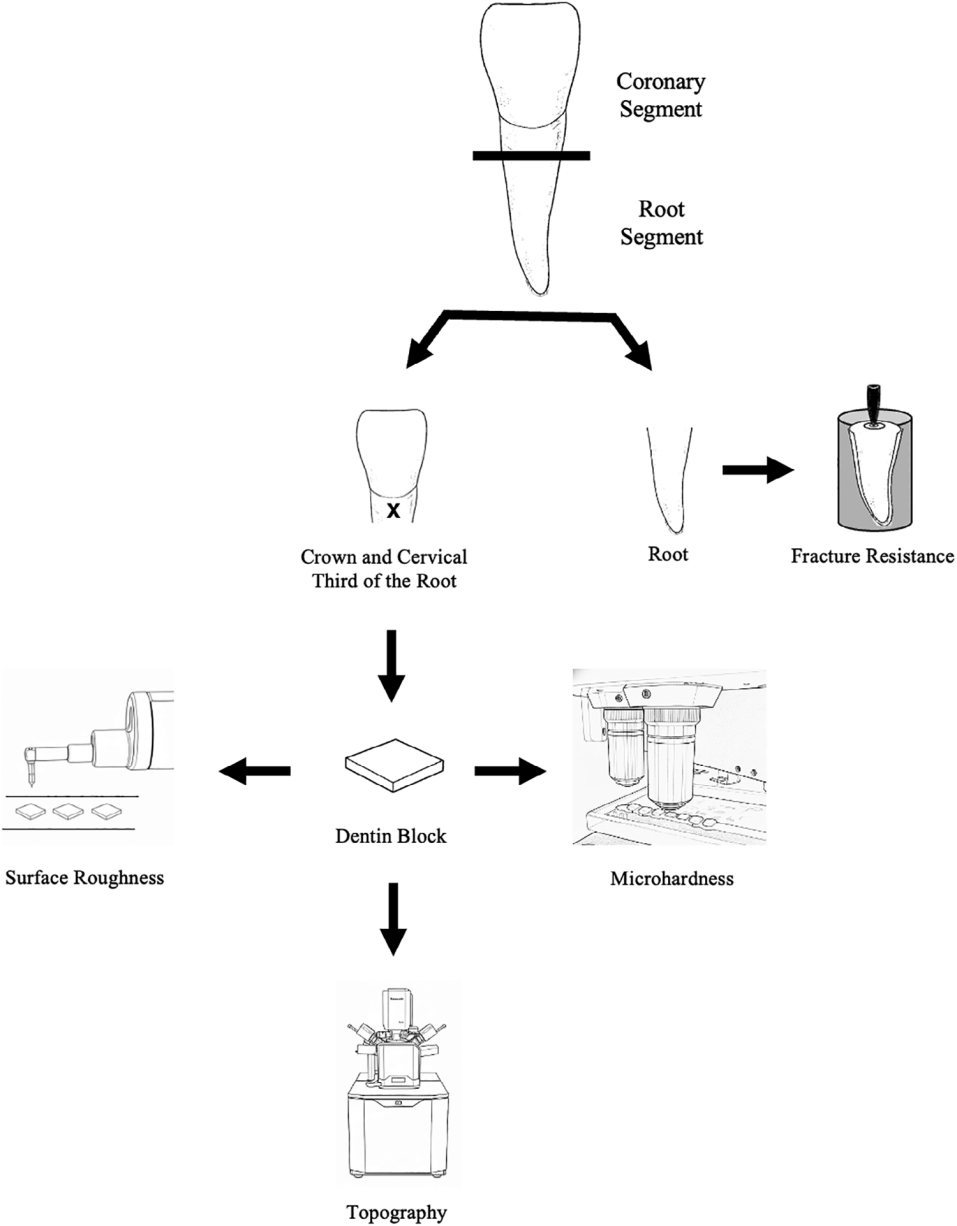
Schematic representation of the sample preparation process and the subsequent analyses performed on each root dentin segment.

The crowns of the teeth were removed using a double‐sided diamond disc (American Burrs) under constant irrigation in a handpiece (Kavo), standardizing the root length to 16 mm (Figure 1). To establish experimental groups with homogeneous and anatomically balanced roots for fracture resistance test, the following preoperative anatomical parameters were recorded: weight (g), mesiodistal (MD) dimension (mm), and buccolingual (BL) dimension (mm). The MD and BL dimensions were measured at the most cervical portion of the root using a calibrated digital caliper. Each sample weight was determined using a precision balance [22].

### Sample preparation

To perform endodontic treatment, the working length was established at 1 mm short of the apical foramen, resulting in a standardized length of 15 mm for all roots. Root canal preparation was performed using the crown‐down technique. Gates‐Glidden burs sizes #4 and #3 (Dentsply Maillefer) were used to prepare the coronal and middle thirds of the canal, respectively. The apical third was instrumented manually with stainless steel files (Dentsply Maillefer) up to size #80. Subsequently, the 84 roots were randomly assigned into six groups (*n* = 14) according to the endodontic irrigant and the number of endodontic treatment visits:

- 0.9% saline solution—single‐visit;
- 0.9% saline solution—two‐visits;
- 2.5% NaOCl—single‐visit;
- 2.5% NaOCl—two‐visits;
- 2% CHX—single‐visit;
- 2% CHX—two‐visits.

Preoperative anatomical parameters were considered for sample allocation to ensure morphologically homogeneous groups.

Each simulated treatment visit consisted of irrigating the root canal with 20 mL of the respective experimental solution for 30 min, with the solution renewed every 3 min. Subsequently, all specimens were irrigated with 5 mL of 17% ethylenediaminetetraacetic acid for 5 min to remove the smear layer, followed by 10 mL of distilled water for 5 min. For groups simulating two‐visits, after the first irrigation protocol, the cervical portion of the root was sealed with a light‐cured temporary restorative material (Bioplic; Biodinâmica Química e Farmacêutica) applied in a single increment and light‐cured for 40 s (Radii Cal; SDI). The samples were then stored in distilled water at 37°C for 1 wk. After this period, the temporary restoration was removed using an explorer probe #5, and the irrigation protocol was repeated to simulate the second visit [17].

### Fracture resistance test and failure mode

Immediately after completing each experimental protocol, the samples were subjected to fracture resistance test. Initially, the roots were wrapped in a 0.2 mm‐thick aluminum foil and positioned in cylindrical plastic molds (20 mm × 25 mm). The molds were then filled with self‐curing acrylic resin up to 2 mm below the cementoenamel junction. After resin polymerization, the aluminum foil was carefully removed and replaced with a thin layer of silicone to simulate the periodontal ligament [22].

All specimens were subjected to static loading using a universal testing machine (Emic DL‐2000; Equipamentos e Sistemas de Ensaio LTDA.). A compressive load was applied to the root using a 6 mm‐diameter stainless steel sphere, positioned centrally under the root canal. The load was applied at a constant crosshead speed of 1 mm/min until failure occurred (complete fracture), and the maximum load in Newtons (N) for each specimen was recorded. For fracture pattern analysis, specimens were examined under a stereomicroscope (Discovery V20; Carl‐Zeiss) at 10× magnification. Fracture types were classified according to Al‐Hiyasat *et al.* [23] as follows: (F1) vertical root fracture aligned with the long axis of the root, resulting in two fragments; and (F2) discontinuous vertical root fracture resulting in more than two fragments.

### Surface roughness test

Dentin blocks were obtained from the buccal surface of the root, specifically from the region corresponding to the cervical third, thereby encompassing the root canal lumen in this area. This portion remained attached to the coronal portion of the teeth during initial sectioning (Figure 1). Therefore, half of these coronary portions (*n* = 42) were randomly selected, and a dentin block measuring 5 mm × 5 mm × 2 mm was prepared from each using a double‐sided diamond disc (American Burrs) under constant irrigation with a handpiece (Kavo). To standardize and smooth the surfaces, the non‐pulpal (enamel) side of each specimen was manually polished with #500 grit silicon carbide paper under water cooling until flat. The pulpal (dentin) surface was then sequentially polished using #1200 and #2000 grit paper for 1 min each. Finally, the polished blocks were placed in an ultrasonic bath with deionized water for 5 min [17].

The pulpal side of all samples was subjected to the same irrigation protocol previously described for the six subgroups (*n* = 7), with the dentin blocks randomly allocated among them. Surface roughness was evaluated using a contact stylus profilometer (Mitutoyo SJ‐410; Mitutoyo Corporation). Measurements were taken at three distinct points along each axis of the sample (*x* and *y*), following the ISO 4287 standard [24]. The mean surface roughness (Ra) and the maximum height from peak to valley (Rz) were recorded.

### Microhardness test

Following surface roughness analysis, the same dentin blocks from each subgroup (*n* = 7) were used for microhardness test. Measurements were performed using a Shimadzu microhardness tester (HMV‐G 20DT) equipped with a pyramidal Vickers indenter (136°) on the pulpal surface of each specimen [25], applying a load of 1 kgf (9.8 N) with a dwell time of 7 s. Three indentations were made in the central region of each dentin block, maintaining a minimum spacing of 100 µm between them to prevent overlap or measurement interference. The indentations were measured using the device's integrated optical system, and the results were expressed as Vickers hardness number. The final microhardness value for each specimen was calculated as the mean of the three measurements.

### Root dentin topography

For topography analysis, two additional dentin blocks were prepared for each group (*n* = 2), following the same procedures described previously. After completion of the irrigation protocols, the samples were dried and sputter‐coated with gold. The pulpal surfaces were then examined using a scanning electron microscope (JSM 6360; JEOL). A qualitative analysis of dentin topography was conducted at ×2000 magnification [17].

### Statistical analysis

Quantitative data were assessed for normality using the Shapiro–Wilk test. Data related to preoperative anatomical factors, fracture resistance, and dentin microhardness followed a normal distribution. In contrast, surface roughness data showed a non‐normal distribution and were therefore subjected to logarithmic transformation to enable the application of parametric statistical tests. Subsequently, all outcomes were analyzed using two‐way anova, with Tukey's post hoc test applied when appropriate (factors: “endodontic irrigants” and “endodontic treatment visits”). Additionally, the distribution of fracture patterns was evaluated using Fisher's exact test. Statistical analyses were performed using SPSS v.26 (IBM) and Statistix v.8.1 (Analytical Software), with the significance level set at 5%.

## RESULTS

### Preoperative anatomical factors

Table 1 presents the mean values of the MD and BL dimensions, as well as the weight of the bovine roots used for the fracture resistance test. No statistically significant differences were found among the groups for these preoperative anatomical factors (*p* > 0.05), confirming the anatomical homogeneity of the samples.

**TABLE 1:** Mean and standard deviation of preoperative anatomical factors mesiodistal (MD) dimension (mm), buccolingual (BL) dimension (mm) and weight (g) of the bovine roots used in the study.

	Endodontic treatment visits		
	Single‐visit		
Endodontic irrigants	MD dimensions	BL dimensions	Weight
Saline solution	6.46 ± 0.83^A^	7.39 ± 0.45^A^	0.83 ± 0.18^A^
2.5% NaOCl	6.50 ± 0.73^A^	7.37 ± 0.72^A^	0.87 ± 0.21^A^
2% CHX	6.48 ± 0.70^A^	7.36 ± 0.60^A^	0.85 ± 0.17^A^

	Two‐visits		
	MD dimensions	BL dimensions	Weight
Saline solution	6.45 ± 0.71^A^	7.38 ± 0.61^A^	0.82 ± 0.20^A^
2.5% NaOCl	6.46 ± 0.55^A^	7.39 ± 0.38^A^	0.84 ± 0.14^A^
2% CHX	6.49 ± 0.43^A^	7.36 ± 0.65^A^	0.83 ± 0.15^A^

*Note*: Equal uppercase letters indicate statistical similarities between groups for each preoperative anatomic factor, considering the two‐way anova test (*p* > 0.05).

Abbreviations: CHX, chlorhexidine; NaOCl, sodium hypochlorite.

### Fracture resistance and fracture patterns

Table 2 presents the fracture resistance values of bovine roots subjected to different experimental protocols. Neither the factor “endodontic irrigants” nor the factor “endodontic treatment visits” significantly influenced the results, indicating statistical similarity among irrigation with saline solution, 2.5% NaOCl, and 2% CHX, regardless of whether a single‐ or two‐visits were simulated (*p* > 0.05). Additionally, the interaction between the two factors also showed no significant effect on fracture resistance (*p* > 0.05).

**TABLE 2:** Mean and standard deviation of fracture resistance (in N) of bovine roots subjected to root canal irrigation with different irrigating solutions, simulating single‐ or two‐visits endodontic treatment.

	Endodontic treatment visits^a^		
Endodontic irrigants	Single‐visit	Two‐visits	Overall^b^
Saline solution	2039.86 ± 431.13^A^	1989.23 ± 309.85^A^	2014.55 ± 369.87^A^
2.5% NaOCl	1995.23 ± 341.30^A^	2148.75 ± 383.44^A^	2071.99 ± 364.67^A^
2% CHX	2086.37 ± 367.59^A^	2147.38 ± 287.90^A^	2116.87 ± 325.47^A^
Overall^c^	2040.49 ± 374.71^A^	2095.12 ± 322.13^A^	

Abbreviations: CHX, chlorhexidine; NaOCl, sodium hypochlorite.

^a^ Equal uppercase letters indicate statistical similarities between groups considering the two‐way anova test (*p* > 0.05).

^b^ Equal uppercases letters in the overall column indicate statistical similarities for the factor “endodontic irrigants,” considering the two‐way anova test (*p* > 0.05).

^c^ Equal uppercases letters in the overall row indicate statistical similarities for the factor “endodontic treatment visits,” considering the two‐way anova test (*p* > 0.05).

**FIGURE 2:**
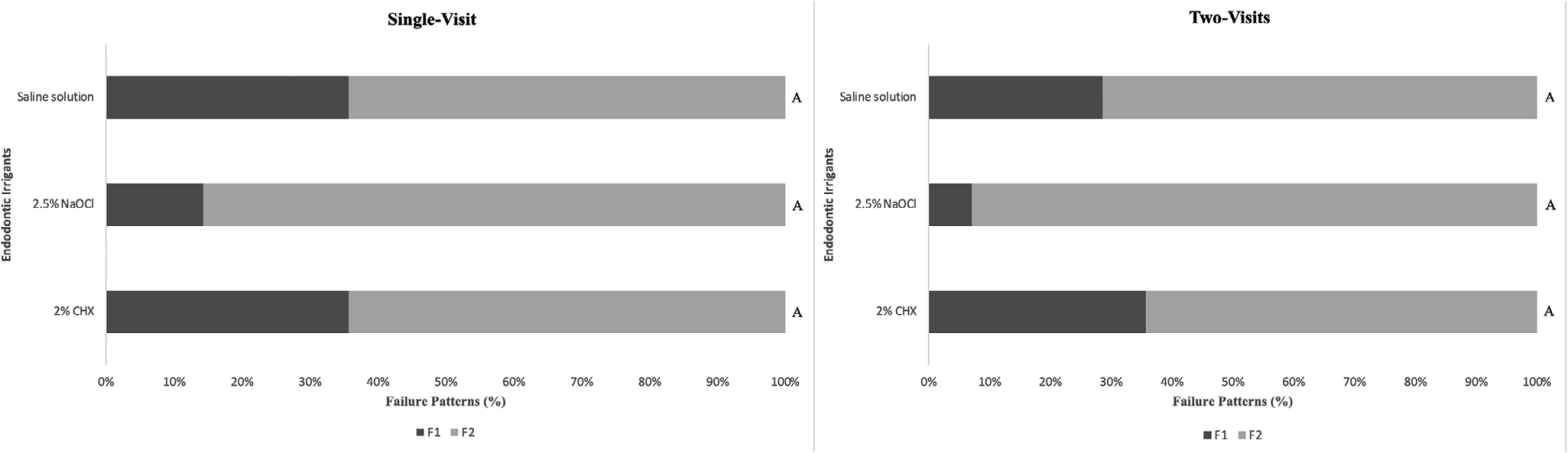
Proportional distribution of fracture patterns (%) in all experimental groups.

Figure 2 shows the distribution of fracture patterns in the bovine roots after fracture resistance test. Overall, 73.81% of the failures were classified as type F2, whereas 26.19% were classified as type F1. There were no statistically significant differences in the distribution of fracture types among the experimental groups (*p* > 0.05).

### Surface roughness

Table 3 presents the surface roughness values (Ra and Rz parameters) of the bovine dentin blocks subjected to the different experimental protocols. The factors “endodontic irrigants” and “endodontic treatment visits” significantly influenced the results (*p* < 0.05). For both Ra and Rz parameters, dentin blocks irrigated with 2% CHX exhibited lower surface roughness values compared to those treated with 2.5% NaOCl (*p* < 0.05), whereas no significant difference was observed for the saline solution (*p* > 0.05). Additionally, simulating two‐visits endodontic treatment resulted in increased surface roughness (Ra and Rz) compared to the single‐visit simulation (*p* < 0.05). However, the interaction between the two factors did not reach statistical significance, although the *p*‐value was close to the threshold (*p* = 0.07).

**TABLE 3:** Mean and standard deviation of surface roughness parameters (Ra and Rz, in µm) for bovine dentin blocks subjected to root canal irrigation with different irrigating solutions, simulating single‐ or two‐visits endodontic treatment.

Endodontic irrigants	Endodontic treatment visits					
	Roughness (Ra)^a^		Overall^b^	Roughness (Rz)^a^		Overall^b^
	Single‐visit	Two‐visits		Single‐visit	Two‐visits	
Saline solution	0.63 ± 0.41^BC^	0.64 ± 0.21^ABC^	0.64 ± 0.32^AB^	3.43 ± 1.87^BC^	3.58 ± 0.98^ABC^	3.50 ± 1.44^AB^
2.5% NaOCl	0.56 ± 0.15^BC^	1.12 ± 0.53^A^	0.84 ± 0.48^A^	3.19 ± 0.83^BC^	5.76 ± 2.14^A^	4.47 ± 2.05^A^
2% CHX	0.36 ± 0.10^C^	0.81 ± 0.27^AB^	0.58 ± 0.30^B^	2.16 ± 0.52^C^	4.12 ± 0.98^AB^	3.14 ± 1.27^B^
Overall^c^	0.52 ± 0.27^B^	0.85 ± 0.40^A^		2.93 ± 1.29^B^	4.49 ± 1.69^A^	

Abbreviations: CHX, chlorhexidine; NaOCl, sodium hypochlorite.

^a^ Equal uppercase letters indicate statistical similarities between groups considering the two‐way anova and Tukey's post hoc tests (*p* > 0.05).

^b^ Equal uppercases letters in the overall column indicate statistical similarities for the factor “endodontic irrigants,” considering the two‐way anova and Tukey's post hoc tests (*p* > 0.05).

^c^ Equal uppercases letters in the overall row indicate statistical similarities for the factor “endodontic treatment visits,” considering the two‐way anova and Tukey's post hoc tests (*p* > 0.05).

### Microhardness

Table 4 presents the Vickers microhardness values of the bovine dentin blocks subjected to the different experimental protocols. The factor “endodontic irrigants” significantly influenced the results (*p* < 0.05), with dentin blocks irrigated with 2% CHX showing lower microhardness values compared to those treated with saline solution or 2.5% NaOCl (*p* < 0.05). In contrast, the isolated factor “endodontic treatment visits” had no significant effect on dentin microhardness (*p* > 0.05). However, the interaction between both factors showed a statistically significant influence (*p* < 0.05).

**TABLE 4:** Mean and standard deviation of Vickers microhardness (in HV1) of bovine dentin blocks subjected to root canal irrigation with different irrigating solutions, simulating single‐ or two‐visits endodontic treatment.

	Endodontic treatment visits^a^		
Endodontic irrigants	Single‐visit	Two‐visits	Overall^b^
Saline solution	56.82 ± 5.43^A^	48.57 ± 5.35^AB^	52.69 ± 6.72^A^
2.5% NaOCl	48.75 ± 6.34^AB^	50.48 ± 3.18^AB^	49.62 ± 4.90^A^
2% CHX	39.71 ± 8.06^C^	47.09 ± 1.91^BC^	43.40 ± 6.81^B^
Overall^c^	48.43 ± 9.58^A^	48.71 ± 3.84^A^	

Abbreviations: CHX, chlorhexidine; NaOCl, sodium hypochlorite.

^a^ Equal uppercase letters indicate statistical similarities between groups considering the two‐way anova and Tukey's post hoc tests (*p* > 0.05).

^b^ Equal uppercases letters in the overall column indicate statistical similarities for the factor “endodontic irrigants,” considering the two‐way anova and Tukey's post hoc tests (*p* > 0.05).

^c^ Equal uppercases letters in the overall row indicate statistical similarities for the factor “endodontic treatment visits,” considering the two‐way anova and Tukey's post hoc tests (*p* > 0.05).

### Root dentin topography

**FIGURE 3:**
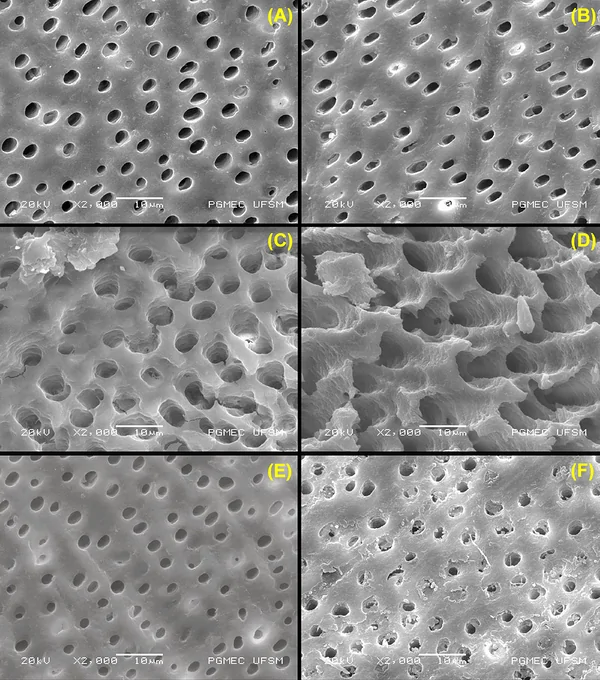
Representative SEM images (×2000 magnification) showing the surface topography of root dentin for each experimental group: (A) 0.9% saline solution—single‐visit, (B) 0.9% saline solution—two‐visits, (C) 2.5% sodium hypochlorite (NaOCl)—single‐visit, (D) 2.5% NaOCl—two‐visits, (E) 2% chlorhexidine (CHX)—single‐visit, and (F) 2% CHX—two‐visits. In (A) and (B), intact dentinal tubules with no evidence of erosion can be observed. In (C) and (D), evident dentin erosion is noted, more pronounced in the two‐visits simulation, characterized by changes in tubule morphology and a reduction of intertubular dentin. In (E) and (F), mild dentin erosion is present, slightly more noticeable in the two‐visits simulation.

SEM images of dentin topography are shown in Figure 3. In the groups treated with 2.5% NaOCl, evident dentin erosion was observed; in the group treated with 2% CHX, minimal erosion was noted; and in the saline group, no significant structural changes were detected. Moreover, in the group simulating two‐visits endodontic treatment, particularly with 2.5% NaOCl, erosion was more pronounced compared to the single‐visit group.

## DISCUSSION

This study simultaneously investigated underexplored endodontic variables regarding their effects on dentin microstructure and, consequently, on dental biomechanical behavior—an important factor for the long‐term survival of endodontically treated teeth. Our results demonstrated statistically significant differences in microstructural outcomes (surface roughness and microhardness), leading to the rejection of both null hypotheses.

The selection of the main irrigant during endodontic treatment remains a topic of debate in endodontics, particularly regarding the antimicrobial efficacy and tissue‐dissolving capacity of the available solutions [6]. Likewise, the decision between single‐ and two‐visits treatment protocols continues to generate discussion, especially concerning the need for intracanal medication, postoperative pain, periapical healing, and overall success rates [15, 26, 27]. However, the literature still lacks evidence and critical analysis on how these variables—the type of irrigant and the number of treatment visits—affect simultaneously dentin microstructure and, consequently, the long‐term survival of endodontically treated teeth. Thus, this study sought to address this gap and provide new insights into the subject.

Our results showed that the use of 2.5% NaOCl resulted in higher dentin surface roughness values than 2% CHX. These findings are likely associated with the topographic alterations observed in the SEM images. The use of 2.5% NaOCl promoted significantly greater dentin erosion compared to 2% CHX, characterized by larger dentinal tubules and reduced intertubular dentin. These results are consistent with previous studies [28, 29] and can be attributed to collagen degradation and consequent damage to the dentin matrix [30]. Conversely, 2% CHX behaved similarly to saline solution in this outcome, indicating that it does not severely affect the dentin surface or generate significant irregularities. Previous studies also report a lesser structural impact of CHX on dentin, supporting our findings [29, 31]. Additionally, the number of endodontic treatment visits influenced this result, as two simulated visits resulted in greater surface roughness compared to a single‐visit. Prolonged exposure to irrigating solutions likely transforms dentin into a porous structure with numerous irregularities in the peritubular and intertubular regions, as well as funnel‐shaped openings of the dentinal tubules [17], reinforcing our observations—particularly in the groups treated with 2.5% NaOCl.

Regarding dentin microhardness, 2% CHX in our study showed reduced values compared to 2.5% NaOCl and saline solution. The effect of CHX on dentin microhardness remains controversial in the literature [32, 33]. Some studies report that CHX can reduce dentin microhardness, possibly due to its interaction with the dentin matrix without promoting mineral deposition, resulting in a less resistant surface [32, 34, 35]. Conversely, other studies suggest that CHX may maintain or even increase dentin microhardness, potentially through the inhibition of matrix metalloproteinases, which are involved in collagen degradation [33, 36]. Despite the evident changes in topography and surface roughness, NaOCl did not affect dentin microhardness, indicating that these alterations did not compromise the dentin ability to withstand mechanical loads [34, 37]. Finally, although no significant difference in dentin microhardness was observed between single‐ or two‐visits treatments alone, the interaction between the type of irrigant and the number of visits was statistically significant, indicating that the effect of the irrigant may vary depending on whether the treatment is performed in single‐ or two‐visits. Therefore, when adopting an optimized protocol, this interaction should be considered to minimize potential damage to the dentin microstructure.

Although the irrigants tested, simulating single‐ and two‐visits endodontic treatments, affected surface roughness, topography, and dentin microhardness, these changes were not sufficient to negatively impact root fracture resistance in our study. Therefore, it is reasonable to assume that microstructural alterations in dentin are not necessarily capable of causing clinically relevant issues, such as tooth fracture. In the simulated scenarios, it is hypothesized that, although dentin was affected, the extent of alteration did not reach a threshold capable of compromising its mechanical integrity or increasing susceptibility to early failure. Previous studies using common chemicals in endodontics have similarly demonstrated that structural damage to dentin does not always translate into reduced fracture resistance, reinforcing our findings [38, 39].

Therefore, it is reasonable to assume that the selection of the main irrigant and the number of treatment visits are not decisive factors regarding the susceptibility of endodontically treated teeth to fracture. Nevertheless, further studies are strongly recommended to simulate more critical clinical conditions and establish a safety threshold considering these variables. Additionally, previous research has shown that prolonged contact of irrigants with dentin may impair the interaction between resin materials and the substrate, reducing adhesiveness at the restorative interface and compromising coronal sealing [17]. In this context, this issue warrants attention for the optimal selection of therapeutic protocols, given the crucial role of coronal sealing in the long‐term success of endodontic treatment [40].

As a limitation of the present study, bovine teeth were used due to their availability and ease of standardization. However, bovine and human dentin share similar mechanical and structural properties [41, 42], and the use of bovine teeth in dental research is a well‐established and widely accepted practice [43], supporting the validity of our findings. Another potential limitation concerns the exclusive use of the root portion in the fracture tests. Nevertheless, this methodological choice has been previously adopted [21] and enabled strict sample standardization, minimizing anatomical confounding factors. Finally, the lack of evaluation of the sequential use of NaOCl and CHX can be considered a limitation of this study, as this combination is also a widely used clinical protocol. However, we emphasize that our research focus was to evaluate the isolated effect and the interaction between the number of simulated endodontic treatment visits and the type of main irrigating solution on the outcomes of interest, rather than to assess a specific clinical protocol.

Considering our findings, the use of 2.5% NaOCl and 2% CHX as irrigants, regardless of whether endodontic treatment is performed in a single or two‐visits, does not increase tooth susceptibility to fracture, showing behavior similar to that of saline solution. However, although these irrigants do not affect this clinically relevant outcome, both solutions, along with the number of visits, significantly influence dentin microstructure, affecting surface roughness, topography, and microhardness. Therefore, future studies are encouraged to assess long‐term treatment outcomes to better understand the behavior of endodontically treated teeth over time in relation to these variables.

## CONCLUSION

In experimental scenarios simulating single‐ and two‐visits endodontic treatments, the evaluated irrigants can promote changes in dentin surface roughness, topography, and microhardness. However, such microstructural modifications did not result in reduced fracture resistance.

## AUTHOR CONTRIBUTIONS

**Conceptualization**: Yasmin Padoin and Guilherme Pauletto. **Data curation**: Yasmin Padoin, Sidnei Flores de Pellegrin, and Duvan Cala Castillo. **Investigation**: Yasmin Padoin, Sidnei Flores de Pellegrin, and Guilherme Pauletto. **Methodology**: Yasmin Padoin, Sidnei Flores de Pellegrin, Duvan Cala Castillo, William Lemos Bevilaqua, Gabriel Kalil Rocha Pereira, and Guilherme Pauletto. **Software**: Yasmin Padoin, Sidnei Flores de Pellegrin, Duvan Cala Castillo, William Lemos Bevilaqua, Gabriel Kalil Rocha Pereira, and Guilherme Pauletto. **Validation**: Yasmin Padoin, Sidnei Flores de Pellegrin, Duvan Cala Castillo, William Lemos Bevilaqua, Gabriel Kalil Rocha Pereira, and Guilherme Pauletto. **Visualization**: Yasmin Padoin, Sidnei Flores de Pellegrin, Duvan Cala Castillo, William Lemos Bevilaqua, Gabriel Kalil Rocha Pereira, and Guilherme Pauletto. **Project administration**: Guilherme Pauletto. **Resources**: William Lemos Bevilaqua, Gabriel Kalil Rocha Pereira, and Guilherme Pauletto. **Supervision**: Guilherme Pauletto. **Formal analysis**: Guilherme Pauletto. **Writing—original draft**: Yasmin Padoin and Guilherme Pauletto. **Writing—review and editing**: Yasmin Padoin, Sidnei Flores de Pellegrin, Duvan Cala Castillo, William Lemos Bevilaqua, Gabriel Kalil Rocha Pereira, and Guilherme Pauletto.

## CONFLICT OF INTEREST STATEMENT

The authors declare no conflicts of interest.

## FUNDING INFORMATION

This study did not receive funding.
